# Genic regions of a large salamander genome contain long introns and novel genes

**DOI:** 10.1186/1471-2164-10-19

**Published:** 2009-01-13

**Authors:** Jeramiah J Smith, Srikrishna Putta, Wei Zhu, Gerald M Pao, Inder M Verma, Tony Hunter, Susan V Bryant, David M Gardiner, Timothy T Harkins, S Randal Voss

**Affiliations:** 1 Department of Biology and Spinal Cord and Brain Injury Research Center, University of Kentucky, Lexington, KY 40506, USA; 2 The Salk Institute for Biological Studies, La Jolla, CA 92037, USA; 3 Department of Developmental and Cell Biology, University of California Irvine, Irvine, CA 92697, USA; 4 The Developmental Biology Center, University of California Irvine, Irvine, CA 92697, USA; 5 Roche Applied Science, Indianapolis, IN 46250, USA; 6 University of Washington, Department of Genome Sciences, Seattle, WA 98195, USA; 7 Benaroya Research Institute at Virginia Mason, Seattle, WA 98101, USA

## Abstract

**Background:**

The basis of genome size variation remains an outstanding question because DNA sequence data are lacking for organisms with large genomes. Sixteen BAC clones from the Mexican axolotl (*Ambystoma mexicanum*: c-value = 32 × 10^9 ^bp) were isolated and sequenced to characterize the structure of genic regions.

**Results:**

Annotation of genes within BACs showed that axolotl introns are on average 10× longer than orthologous vertebrate introns and they are predicted to contain more functional elements, including miRNAs and snoRNAs. Loci were discovered within BACs for two novel EST transcripts that are differentially expressed during spinal cord regeneration and skin metamorphosis. Unexpectedly, a third novel gene was also discovered while manually annotating BACs. Analysis of human-axolotl protein-coding sequences suggests there are 2% more lineage specific genes in the axolotl genome than the human genome, but the great majority (86%) of genes between axolotl and human are predicted to be 1:1 orthologs. Considering that axolotl genes are on average 5× larger than human genes, the genic component of the salamander genome is estimated to be incredibly large, approximately 2.8 gigabases!

**Conclusion:**

This study shows that a large salamander genome has a correspondingly large genic component, primarily because genes have incredibly long introns. These intronic sequences may harbor novel coding and non-coding sequences that regulate biological processes that are unique to salamanders.

## Background

It was established before the advent of DNA sequencing that organisms show incredible variation in genome size. This presented a paradox because scientists originally expected a positive relationship between genome size and organism complexity [1]. The paradox was partially resolved by partitioning overall genome size into two compartments: protein coding vs non-protein coding. This partition showed that organisms tend to have similar numbers of genes but non-coding and presumptively non-functional portions of genomes vary greatly [2]. In recent years, perception has changed; it is well-established that non-genic regions of genomes encode regulatory and structural information, and functional RNAs [3,4]. Surprisingly, almost all of the genome is transcribed in some organisms, not simply the protein-coding portion [5-7]. Some repetitive sequence classes that were thought to only selfishly expand genome size at the expense of the host are known to regulate transcription and contribute to gene evolution [8-12]. Genomes contain large non-coding regions that are conserved across species [13-15], and lineage-specific, non-coding DNA between distantly related species is associated with the same regulatory functions; such patterns are consistent with non-coding DNA having a regulatory function [16,17]. Finally, the amount of non-coding DNA does scale with developmental complexity in some comparative studies [18]. These findings are motivating renewed interest into genome diversity and function. Unfortunately, DNA sequence data are completely lacking for organisms with large genomes.

In this study, 454 DNA sequencing was used to obtain the first glimpse of a salamander genome. The Mexican axolotl (*Ambystoma mexicanum*) was selected because it is a model organism with an average-sized salamander genome: ~32 × 10^9 ^bp distributed among 14 haploid chromosomes [19]. Considering the possibility of extensive repetitive DNA tracts in the axolotl genome that would confound downstream sequence assembly, it was reasoned that genic regions of the genome would be less likely to contain repetitive DNA. Also, recent analyses suggest that regulatory elements within the human genome tend to be associated with the location of known genes [20]. Thus, a partial BAC library was developed and PCR screening identified 16 clones that contain expressed sequence tags (ESTs) [21]. This allowed direct comparison of orthologous genic regions between axolotl and the human genome and analysis of two BACs that contained presumptively novel axolotl transcripts. To complement this approach, computational analyses were used to search existing EST databases for genes that are specific to axolotls and perhaps other amphibians. The results from these analyses, discussed below, begin to address the basis of the axolotl's large genome size and the significance of excess DNA in genic regions.

## Results

### BAC sequence assembly and annotation

A small BAC library (36,864 clones) was constructed and screened by PCR to identify 16 clones that contained coding sequences for previously identified ESTs (Table 1). Altogether, these clones span more than 1.7 megabases (non-redundant) of the axolotl genome. BAC clones were end-sequenced using the ABI-Sanger method and then 454 sequencing technology was used to generate several thousand, high quality sequence reads for each clone (Table 2). Sequence assembly statistics (N50 and average sequence coverage) indicate that high quality assemblies were generated for each BAC; seven BAC assemblies yielded a single long contig and three BAC assemblies yielded two contigs separated by single gaps. The sequence coverage provided by the assemblies approximated the estimated size of BAC clones on agarose gels (data not shown). The remaining assemblies consisted of 6 or fewer large contigs. The reason why a few contigs yielded incomplete assemblies is not clear because different numbers of high quality reads were obtained for each BAC and contig numbers within assemblies were not correlated with sequencing depth. However, in only one case was it clear (while editing and annotating contigs) that repetitive sequences confounded contig assembly of a BAC (clone H3_4F24). Indeed, very few repetitive DNA sequences were identified overall within axolotl BACs, with retrotransposons representing the largest fraction (Table 3). These results suggest that genic regions of the axolotl are not completely structured by repetitive sequences. Annotated BAC assemblies have been deposited in GenBank [GenBank: EU686400–EU686415].

**Table 1:** Identity and structure of genes within BACs

**BAC**	**Ambystoma Sequence**	**Presumptive Human Ortholog**	**Complete Axolotl** **ORF?**	**Introns** **Identified**	**Exons** **Identified**
H3_1D2	Tig_NM_4343_Contig_1	NP_004334.1 calreticulin precursor	Complete	8	9

H3_4A11	Mex_Nohits_2574_Contig_1	Unknown	Complete	9^c^	10

H3_4F24	Tig_NM_362_Contig_1	NP_003247.1 tissue inhibitor of metalloproteinase 4 precursor	Unknown	1^c^	3^e^

H3_37I11	Tig_NM_7006_Contig_1	NP_008937.1 cleavage and polyadenylation specific factor 5	Partial	5	6

H3_37I23	Tig_NM_18948_Contig_1	NP_061821.1 mitogen-inducible gene 6 protein	Complete^a^	-	1

H3_37N9	Mex_Nohits_697_Contig_1	NP_071436.1 platelet receptor Gi24	Partial	5	5

H3_41L21	Mex_NM_687_Contig_1	NP_000678.1 S-adenosylhomocysteine hydrolase	Pseudogene^b^	-	-

H3_46H10	Tig_NM_1428_Contig_1	NP_001419.1 enolase 1	Partial	-	1

H3_48F8	Tig_NM_859_Contig_1	NP_000850.1 3-hydroxy-3-methylglutaryl-Coenzyme A reductase	Complete	14	15

H3_48K23	Mex_NM_20169_Contig_4	NP_996846.1 retinoic acid receptor responder	Partial	1^c^	2

H3_61C19	Mex_Nohits_221_Contig_2	Unknown	Complete	-	1

H3_61K9	Mex_NM_5032_Contig_2	NP_005023.2 plastin 3	Partial	1	2

H3_62O21	Mex_NM_18947_Contig_1^f^	NP_002550.2 purinergic receptor P2X3	Partial	7	8

H3_67L15	Tig_NM_4343_Contig_1	NP_004334.1 calreticulin precursor	Complete	8^e^	9

H3_71A8	Tig_NM_182513_Contig_1	NP_872319.1 spindle pole body component 24 homolog	False positive	-	-

H3_71D15	Mex_NM_6276_Contig_2	NP_001026854.1 splicing factor, arginine/serine-rich 7, 35 kDa	Complete^a^	-	1

a – start codon was not identified, but it is likely present in assembled sequence. b – this BAC contains a presumptive processed pseudogene. The aligning BAC and cDNA sequence are 91% identical and the BAC sequence contains no introns. c – not used in multispecies alignments due to lack of obvious vertebrate orthologies. d – exons were identified on two different contigs. e – not considered in multispecies alignments due to redundancy with H3_1D2. f – contig was originally identified as cytochrome c.

**Table 2:** Summary statistics for axolotl BAC sequencing and assembly

**Total Assembly Length (bp)**	**Number of Contigs**	**N50 Length (bp)**	**Sequences Covering BAC**	**Avg. Seq. Coverage**
102210	1	102210	9777	24.51
137463	1	137463	11218	20.27
123412	6	21185	9033	23.44
113164	2	56755	9036	22.39
137255	4	51165	9238	12.78
118832	2	51165	8882	18.57
117549	1	117549	9049	19.83
120452	3	48654	5323	12.76
120467	1	120467	6120	13
125197	6	31049	2093	6.55
99252	2	51540	7858	19.82
102224	1	102224	6448	16.08
113103	1	113103	7360	15.99
110421	3	67330	3833	9.01
114195	4	41502	12090	27.65
108550	1	108550	7159	16.58

**Table 3:** Percentages of repetitive elements within BACs and introns

	**Axolotl**		**Human**
		
	**BACs**	**Introns**	**Introns**
Total interspersed:	2.32	1.82	0.38
			
Total retroelements:	2.24	1.72	0
SINEs:	0	0	0
LINEs:	0.29	0.25	0
L2/CR1/Rex	0.11	0.16	0
R1/LOA/Jockey	0	0	0
RTE/Bov-B	0.01	0.01	0
L1/CIN4	0.18	0.07	0
LTR elements:	1.95	1.47	0
Gypsy/DIRS1	1.42	0.78	0
Retroviral	0.24	0.37	0
			
DNA transposons:	0.08	0.1	0.38
Hobo-Activator	<0.01	0.05	0
PiggyBac	0	0	0.18
Tourist/Harbinger	0.06	0.04	0

### Introns and exons within BACs

To further investigate the structure of genic regions within the axolotl genome, introns and exons were identified within BACs and compared to orthologous sequences from humans. BLAST analysis confirmed the presence of targeted EST sequences within 14 of 16 BAC assemblies. The length of orthologous coding sequences between axolotl and humans is highly conserved, as is the location of exon/intron boundaries (Table 1; Additional file 1). However, axolotl introns are strikingly longer than human introns: within five genes for which orthology could be firmly established, axolotl introns average 9454 bp while human introns average only 1938 bp (N = 32 introns compared). Further comparisons show that axolotl introns are approximately 14× larger than orthologous introns from chicken (N = 32) and 12× larger than orthologous introns from *Xenopus tropicalis *(N = 25; purinergic receptor P2X3 was not identified in the *X. tropicalis *assembly) (Additional file 1, Figure 1). Thus, non-coding genic regions are contributing significantly more to axolotl genome size than they are to vertebrates with "average-sized" genomes.

**Figure 1:**
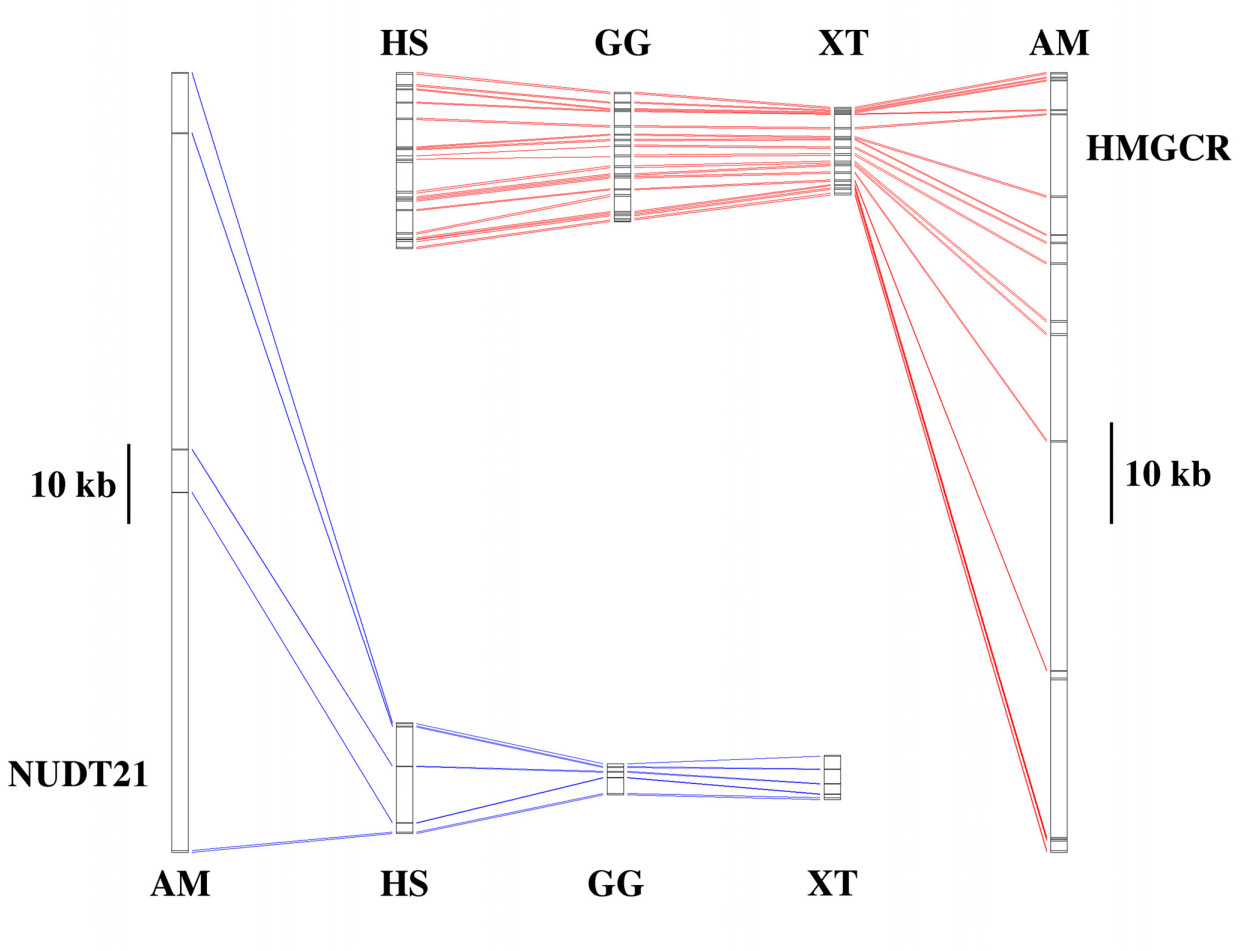
**Comparison of intron lengths among the axolotl (AM), human (HS), chicken (GG), and frog *Xenopus tropicalis *(XT) for *cleavage and polyadenylation specific factor 5 *(NUDT21) and 3-hydroxy-3-methylglutaryl-Coenzyme A reductase (HMGCR)**. One exon of HMGCR could not be identified in the *X tropicalis *genome.

### Composition of axolotl introns

It is possible that axolotl introns are large because they contain DNA sequence classes that are unique or over-represented in comparison to other vertebrates. To test this idea, all axolotl introns and orthologous human introns were searched for self-similarity, repetitive DNAs (transposons and retrotransposons), and non-coding RNAs (including miRNAs and snoRNAs). Examination of individual self-self intron alignments and alignment of the concatenated intron dataset revealed that axolotl introns do not contain extensive tracts of repetitive DNA and are composed of largely unique sequence (Additional files 2 and 3). Multiple retroelement types were identified in axolotl introns in the selected genes but none were identified in the orthologous human introns (Table 3). Although the human genome contains many repeat classes, the only repeats identified in this sample of human introns were DNA transposons. The proportion of nucleotides accounted for by interspersed repetitive sequences is significantly higher in axolotl introns, relative to human introns (1.82% *vs*. 0.38%, Z = 25.6, p << 0.0001). A total of 70 candidate miRNA precursors and 21 snoRNAs (16 HACA type snoRNAs and 5 CD type snoRNAs) were identified from sense DNA strands of the axolotl (Additional files 4 and 5). The miRNAs totaled 7 kb and the snoRNAs totaled 2.7 kb for a total contribution of 2.7% to overall intron length. By way of comparison, computational searches of 39 orthologous human introns (58,313 bp) identified 6 candidate miRNAs, 1 CD type snoRNA, and no candidate HACA type snoRNAs (Additional files 4 and 5); none of these human introns contain annotated miRNAs or snoRNAs within the current human genome assembly [22]. Thus, the density of predicted small, intronic ncRNAs is significantly higher in axolotls than in humans (Table 4). These analyses show that axolotl introns contain a greater diversity of transposable elements and potentially functional DNA sequence elements than human introns.

**Table 4:** Densities of predicted non-coding RNAs identified within salamander BACs and human orthologous introns

**Predicted ncRNAs**	***Ambystoma***	**Human**	**Z**	**P-value**
miRNA	1.6%	1.0%	11.1	<<1e-4
snoRNA	0.6%	0.1%	15.0	<<1e-4
Total	2.3%	1.2%	17.4	<<1e-4

**Figure 2:**
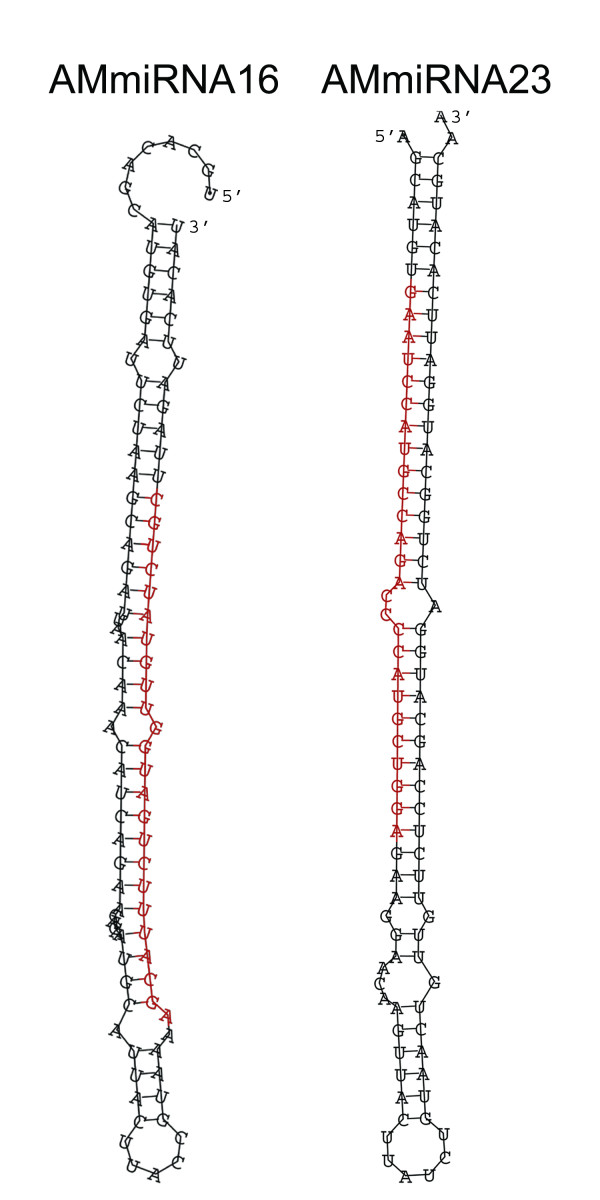
**Structure of two *A. mexicanum *miRNAs (AMmiRNA16, AMmiRNA23) that were predicted from axolotl introns**. The red bases indicated positions where the predicted miRNA sequences show complete identify to small RNAs isolated from regenerating limbs.

The high density of predicted miRNA structures within axolotl introns could be an artifact of the methods that were used to identify candidate miRNAs, or could represent other complex hairpin sequences that do not enter miRNA processing. To investigate this further, predicted miRNA sequences were aligned to 773,450 small RNA sequences that were recently characterized from amputated and regenerating axolotl limbs (unpublished data). This new axolotl miRNA database will be described elsewhere. Two of the predicted miRNAs from axolotl introns had stem regions that aligned perfectly with mature miRNA sequences from the axolotl limb miRNA database (Figure 2): AMmiRNA16 aligned to a single 24 bp sequence and AMmiRNA23 aligned to three independently sampled 26 bp sequences. These perfect alignments suggest that some of the predicted elements within axolotl introns are likely to be bona fide miRNA genes.

### Novel genes

**Figure 3:**
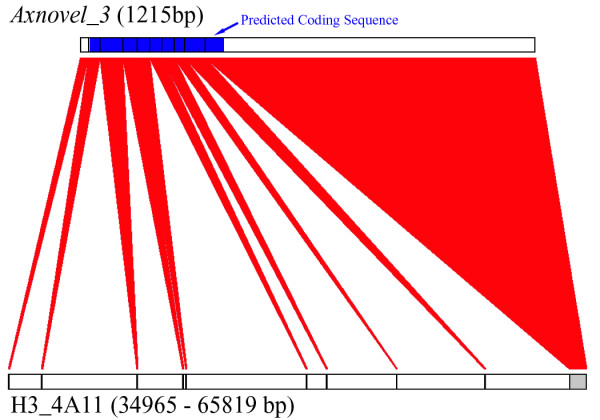
**Intron-exon structure of a novel axolotl salamander gene (*AxNovel_3*) discovered within BAC H3_4A11. Intron/Exon boundaries are represented by vertical black bars**. The predicted coding sequence for *AxNovel_3 *is shaded in blue. Red figures join the relative locations of sequences in the transcript and genomic sequence.

Two of the BACs in this study were selected because they contain transcripts with no known homolog in other vertebrates (Table 1). Results from microarray analyses predict a role for these "no-hit" EST contigs in two unique salamander developmental processes: metamorphosis and regeneration. The no-hit transcript that is encoded on H3_4A11 (Mex_Nohits_2574_Contig_1) is significantly downregulated during spinal cord regeneration, while the no-hit transcript that is encoded on H3_61C19 (Mex_Nohits_221_Contig_2) is significantly upregulated during spinal cord regeneration and downregulated during skin metamorphosis [23,24]. Although some no-hit ESTs are truncated versions of known genes, it is possible that many of the ~2000 no-hit transcripts in the Ambystoma EST database correspond to novel axolotl genes. Annotation of axolotl no-hit EST/BAC alignments supports the later hypothesis. Two novel genes, *Axnovel_1 *and *Axnovel_2*, were identified within H3_4A11 and H3_61C19, respectively. These novel genes correspond to the no-hit transcripts described above. Unexpectedly, a group of no-hit ESTs aligned to a second region of H3_4A11 that is distinct from *Axnovel_1*. These alignments predict a third novel gene (*Axnovel_3*) that has introns and is spliced (Figure 3). None of these three genes show sequence similarity to any known vertebrate gene.

To determine if these novel genes encode proteins or non-coding RNAs, EST/BAC sequence alignments were manually curated and searched for open reading frames (ORFs) using ORF finder at NCBI [25]. In all three cases the longest ORF was oriented 5' to 3' relative to the EST sequences. *Axnovel_2 *and *Axnovel_3 *can be translated into long ORFs (*Axnovel_2 *– 786 bp and *Axnovel_3 *– 360 bp) that are initiated with a start methionine and terminated by a stop codon. Manual curation of *Axnovel_3 *revealed several small exons that were not identified by automated sequence alignments (Figure 3). The coding sequence spans eight small 5' exons that range in length from 21 to 60 bp and extends 48 bp into a longer 3' exon that contains the presumptive 3' UTR of this gene. Nearly the entire length of *Axnovel_1 *(249 of 313 bp) can be translated into a single ORF with a stop codon. The only in-frame methionine codon is located in the middle of the ORF, however the first codon of the longest ORF is CTG, so it is possible that this gene uses an alternative CUG start codon [26]. Interestingly, orthologous EST sequences have also been sampled for *Axnovel_1 *in *A. tigrinum tigrinum*, a close relative. The *A. t. tigrinum *contig shares >98% nucleotide identity with *Axnovel_1 *and also encodes a 5' CUG. It is unclear if *Axnovel_1 *is translated into a functional protein or if it functions as a ncRNA; however maintenance of gene structure and sequence identity between salamander species that diverged several million years ago supports the idea that it is functional.

The most likely mechanism for the origin of novel, functional genes in the *Ambystoma *genome is gene duplication, as there is no evidence for whole genome duplication in *A. mexicanum*. It is important to consider the possibility that the large *Ambystoma *genome may have been shaped by a higher rate of gene duplication and fewer gene losses, and thus contain a greater overall number of genes. If paralogous loci are abundant in the *Ambystoma *genome, then many salamander genes are expected to show relatively more, many-to-one orthology relationships with genes from other vertebrates. To test this hypothesis, paralogs were predicted for a high quality, human-salamander ortholog dataset (N = 577), wherein primary axolotl orthologs were required to cover > 89% of the annotated length of each primary human ortholog. Approximately 86% (N = 498) of the human-axolotl gene pairs in this dataset were predicted to be 1:1 orthologs (Additional file 6). Many: many ortholog groups were predicted for 15 human-axolotl gene sets (Additional file 7) and include members from gene families that are notorious for gene duplication and gene conversion events (e.g. globins, tubulins, and actins). Of the remaining gene pairs, 2.6× more paralogs were predicted for axolotl primary orthologs (Additional files 8 and 9). Specifically, one or more human paralogs correspond to 25 human primary orthologs, yielding 32 different paralogs overall. In comparison, 39 primary axolotl orthologs correspond to 84 different axolotl paralogs. The list of axolotl specific paralogs include *annexin A1 *(N = 4), *ferritin heavy polypeptide *(N = 4), *H3 histone family 3A *(N = 3), *calmodulin 2 *(N = 2), and *matrix metalloproteinase 1 *(N = 2). The largest number of axolotl paralogs (N = 28) was identified for *paternally expressed 10 isoform RF1 *(*peg10*), an imprinted mammalian gene that shows sequence similarity to retrotransposons. As these axolotl paralogs exhibit higher sequence similarity to fish *pol *polyproteins [27] than human *peg10*, they probably correspond to an active retrotransposon family in the axolotl genome. Overall, these data predict 2% more duplicated loci in the axolotl genome versus the human genome (39/577 *vs*. 25/577), and more paralogs are predicted on average for axolotl duplicated loci (2.3 *vs*. 1.3). These estimates support the hypothesis of more lineage specific genes in the axolotl genome than the human genome. Assuming these genes also contain longer introns, the genic portion of the axolotl genome is predicted to exceed the total genome size of some vertebrates (see below).

## Discussion

Comparative DNA sequence data are needed from large genomes to better understand structural and functional features that influence genome size evolution. This study demonstrates that DNA sequence data can be sampled efficiently from the large genome of the Mexican axolotl using 454 DNA sequencing. It was possible to assemble *de novo *short-DNA sequence reads (50–300 bp) from shot gun sequenced BACs into complete contigs, and then use this information to reveal the structure of genic regions of the genome. The results show that axolotl genic regions encode novel genes and make a significant contribution to genome size. In particular, axolotl introns are 5–10× longer than introns in other vertebrates and this maybe typical of salamander genomes [28].

Many different ideas have been proposed to explain genome size variation among organisms. The simplest explanation is a change in the ratio of DNA that codes for proteins versus non-protein coding DNA [29]. Although variation in gene number maybe important, this distinction is too simple because non-protein coding DNA has been shown in recent years to encode a diversity of functional elements. For example, protein-coding sequences (exons) are associated with introns that encode a diversity of regulatory elements and non-coding RNAs that affect transcription, translation, and chromatin structure [30-32]. In order to understand the relationship between genome size and regulatory complexity, it is therefore critical to consider the proportion of DNA that resides in transcribed (genic) versus non-transcribed (i.e. intergenic) DNA. Changes in genome size that occur over relatively short evolutionary timeframes may not result in a correlated expansion of genic regions (i.e. introns), presumably due to greater evolutionary consrtaint [33,34]. However, positive correlations are observed between genome size and the number and length of introns at a broader evolutionary scale [35-37]. Correlations observed at this broader scale are presumably the outcome of drift and selection as population sizes and functional constraints fluctuate over millions of generations [37]. Salamanders are particularly interesting in this regard because they present a situation wherein large genomes are the rule rather than the exception. Very large genomes have likely been maintained within this group at least since the divergence of the ancestral salamander lineage >160 million years ago [38,39]. Thus, salamanders can provide novel insight into the evolutionary potential of vertebrate genomes over deep, evolutionary time.

At this point we can only speculate about the reasons why large introns evolved in *A. mexicanum*. In general, introns tend to be longer in genes that have tissue specific or developmentally relevant functions, than introns in house keeping or widely expressed genes [40-42]. This pattern may reflect evolution of complex transcriptional regulatory mechanisms [43-46]. It is possible that salamanders maintain large introns in-part because they encode information necessary to accomplish unique developmental processes. In particular, salamanders are capable of complex tissue regeneration, and a single genome can express both a metamorphic and paedomorphic outcome [47,48]. These processes involve transcriptional activation and silencing of thousands of genes that may depend upon transcriptional binding sites and ncRNAs within introns. That large salamander introns might have a functional role is supported by the absence of shared repetitive sequences among introns and the prediction of numerous miRNA and snoRNA genes in axolotl introns. It is also possible that long introns indirectly moderate cellular and developmental processes by influencing transcription and mitotic rates [49,50]. We note that the predicted repetitive DNAs and ncRNAs only account for a small proportion of total intron size. Characterization of additional axolotl genes, and in particular genes that function in regeneration and metamorphosis, will help optimize searches for other functional and structural elements (e.g. matrix attachment sites or unknown functional classes) that are associated with large intron size, including "junk" DNA.

## Conclusion

Results from this study show that the genic compartment of the *Ambystoma *genome is incredibly large. Our analysis suggests that genes in the axolotl genome are 5× larger than they are in humans and conservative estimation of lineage specific genes predicts more genes in the salamander genome than the human genome. If there are approximately 2% more genes in the axolotl genome than a 20,000 gene estimate for the human genome, and each salamander gene is on average 5× larger than a 27 kilobase average estimate for human genes [51], the genic portion of the *Ambystoma *genome is estimated to be a staggering 2.8 gigabases! Thus, the large salamander genome is not simply large because of excess, repetitive DNA; the genic component is also correspondingly large. Equally staggering is the fact that our estimate of genic content only accounts for 1/12^th ^of the total genome size of 32 gigabases. Even if considerably more genes are discovered to be novel in the axolotl genome, using more aggressive computational approaches to identify highly divergent proteins, this is not likely to solve the mystery of large genome size in salamanders. Additional, DNA sequencing is needed to solve this mystery and this study shows that new sequencing technologies allow such datasets to be readily generated for organisms with large genomes.

## Methods

### BAC library construction and screening

A BAC Library was constructed from partially digested and size selected genomic DNA that was isolated from the erythrocytes of a single *A. mexicanum *female. Methods for DNA isolation and BAC library construction followed [52]. 36,864 colonies were robotically picked into ninety-six 384 well plates. BAC pools were constructed and screened by PCR with 96 EST primer sets to identify 16 BACs that contained protein-coding loci.

### Sequencing, assembly, and annotation

DNA was isolated for each of the 16 BAC from 200 ml of overnight culture using a Plasmid Maxi Kit (Qiagen). All of the BACs were sequenced in a single 454 GS20 sequencing run, on one plate that was divided into subregions using a sixteen-lane gasket. Clones were also end sequenced using BigDye 3.1 chemistry and electrophoresis on an ABI capillary sequencer. Sequences were screened by *Crossmatch *[53] to remove vector (pCC1BAC), contaminating *E. coli *sequences (NC_002695.1), and additional contaminating sequences (gis:146575, 215104, 469217, 520486, 2501752) that were identified by a preliminary search of all BAC sequences against the NCBI nr database. After automated assembly using *Phrap *[53] (force level = 1, all other parameters set to default), all contigs over 10 kb were aligned to one another to identify contigs that contained presumptively overlapping sequence. These were visually inspected using *SeqMan *(DNASTAR Lasergene) and manually joined when appropriate. Contiguous sequences of assembled BACs were searched (*blastn*) [54] against the complete set of all known salamander transcripts at Sal-Site [55] and human RefSeq (*blastx *and *tblastp*) [56] to identify and annotate gene regions within BACs. For multispecies comparisons, the locations of orthologous intron breaks were identified by aligning (*blat*) [57] human RefSeq proteins to genome sequence of human (Build 36.2), chicken (galGal3), *X. tropicalis *(xenTro2), and salamander (current study). Self/self and all *vs*. all alignments of salamander intron sequences were performed using the program *dottup *(EMBOSS package) [58].

### Identification of repetitive elements and candidate miRNAs and snoRNAs

427,188 bp of sequence from 48 axolotl introns was searched using several algorithms. Salamander BACs, predicted introns, and orthologous introns were searched for known repetitive elements using *RepeatMasker *[59] and libraries of known repeat elements [60]. Candidate miRNAs and snoRNAs were identified on the basis of predicted structural motifs within intronic regions of BACs. To identify candidate miRNAs, BAC sequences were searched using the program *ProMiR II *[61]. These candidate miRNAs were further tested for probable functionality using the program *MiPred *[62]. Candidate snoRNAs were identified using CDSeeker and ACASeeker functions of *snoSeeker *[63], with "modified site" files containing known human methylation and pseudouridine sites.

### Identification of orthologous and paralogous genes

Reciprocal best-Blastx searching between the *Ambystoma *EST assembly and human RefSeq databases identified primary ortholog pairs between *A. mexicanum *and human. To ensure that axolotl EST contigs yielded a high quality dataset for paralog prediction, the analysis was limited to 577 ortholog pairs in which primary *A. mexicanum *orthologs were required to cover >89% of the annotated length of each primary human ortholog. This limited the analysis to relatively short genes whose overall lengths have been conserved during evolution. The axolotl EST contigs were assembled previously using PaCE [64] and CAP3 [65] using a 90% nucleotide identity threshold to assemble ESTs into contigs [21]. Singleton contigs were excluded from the analysis. Orthologous relationships were determined using an informatics approach followed by manual annotation. The conservative *Inparanoid *approach [66,67] was used first to identify presumptive orthologs and paralogs. Primary *A. mexicanum *orthologs were searched (*blastx*) against all *A. mexicanum *contigs [55] and human primary orthologs were searched (*blastx*) against all human RefSeq entries [56]. If these within species searches identified amino acid sequences that were more similar to the primary ortholog, relative to the between species comparison, they were tentatively considered lineage specific paralogs. This parsed human/*A. mexicanum *primary orthologs among 1:1, 1: many, many: 1, or many: many orthology classifiers. This analysis predicted 178 axolotl paralogs and 62 human paralogs. To complement the *Inparanoid *approach, *A. mexicanum *primary orthologs were searched (*blastn*) against EST contigs that have been assembled for a close relative: *A. t. tigrinum*. If 2 or more *A. t. tigrinum *genes were found to reciprocally best match a primary *A. mexicanum *ortholog and significantly similar *A. mexicanum *contigs, the *A. mexicanum *primary ortholog was considered a duplicated locus and the corresponding *A. mexicanum *contigs were considered paralogs. This approach verified 19 of the *Inparanoid *predictions for axolotl paralogs and suggested 18 novel paralogs that were not predicted by *Inparanoid*. Because the approaches described above do not: 1) differentiate paralogs from splice variants, 2) evaluate the quality of EST contigs, or 3) identify all paralogs, it was necessary to manually inspect the quality of all overlapping sequence alignments for presumptive 1:1 orthologs and paralogs. As a result of manual annotation, approximately 50% of the predicted human and axolotl paralogs were discarded and 4 of the 1:1 orthologs were re-classified as duplicated axolotl loci (*anterior gradient 2 homolog*, *integral membrane protein 2b*, *stress-associated endoplasmic reticulum protein 1*, and *parvalbumin*). We note that axolotl gene sequences in this analysis are substantially under sampled by comparison to the known list of human gene sequence. This sampling difference is expected to yield a minimum estimate of the true abundance of axolotl/amphibian paralogs.

## Abbreviations

BAC: bacterial artificial chromosome; EST: expressed sequence tag; miRNA: micro RNA; snoRNA: small nucleolar RNA; ncRNA: non-coding RNA; ORF: open reading frame.

## Authors' contributions

JJS and SRV drafted the manuscript. All authors participated in acquisition, analysis, and interpretation of the data. All authors participated in critical revision of the manuscript.

## Supplementary Material

Additional file 1 **Comparative data for exons and introns.** A table containing exon alignment statistics and corresponding intron lengths for salamander, human, chicken, and frog. Click here for file

Additional file 2 **Self-self sequence alignments.** Plots showing representative self-self sequence alignments for the 4 longest introns that were isolated from *A. mexicanum*. Click here for file

Additional file 3 **Alignment of all salamander introns.** A plot showing the self-alignment of concatenated intronic sequence sampled from *A. mexicanum*. Click here for file

Additional file 4 **miRNAs predicted for axolotl and human.** A table containing positional and statistical information for miRNAs predicted for axolotl and human. Click here for file

Additional file 5 **snoRNAs predicted for axolotl and human.** A table containing positional and statistical information for snoRNAs predicted for axolotl and human. Click here for file

Additional file 6 **Human-axolotl 1: 1 orthologs.** A table containing identities and alignment summary statistics for human-axolotl 1: 1 orthologs. Click here for file

Additional file 7 **Human-axolotl many: many paralogs.** A table containing identities and alignment summary statistics for human-axolotl many: many paralogs predicted for both human and axolotl. Click here for file

Additional file 8 **Human-axolotl many: 1 paralogs.** A table containing identities and alignment summary statistics for human-axolotl many: 1 paralogs predicted for both human and axolotl. Click here for file

Additional file 9 **Human-axolotl 1: many paralogs.** A table containing identities and alignment summary statistics for human-axolotl 1: many paralogs predicted for both human and axolotl. Click here for file
