# Supplementary material for: Genic regions of a large salamander genome contain long introns and novel genes
Source: BMC Genomics. 2009 Jan 13;10:19. doi: 10.1186/1471-2164-10-19 (PMC2633012; doi:10.1186/1471-2164-10-19)

**Additional File 2 –** Plots showing representative self-self sequence alignments for the 4 longest introns that were isolated from *A. mexicanum*. The X and Y axes represent the position along the sequence. The relative location of all alignments >10bp are shown. Repeat sequences should appear as diagonal lines off of the primary diagonal.


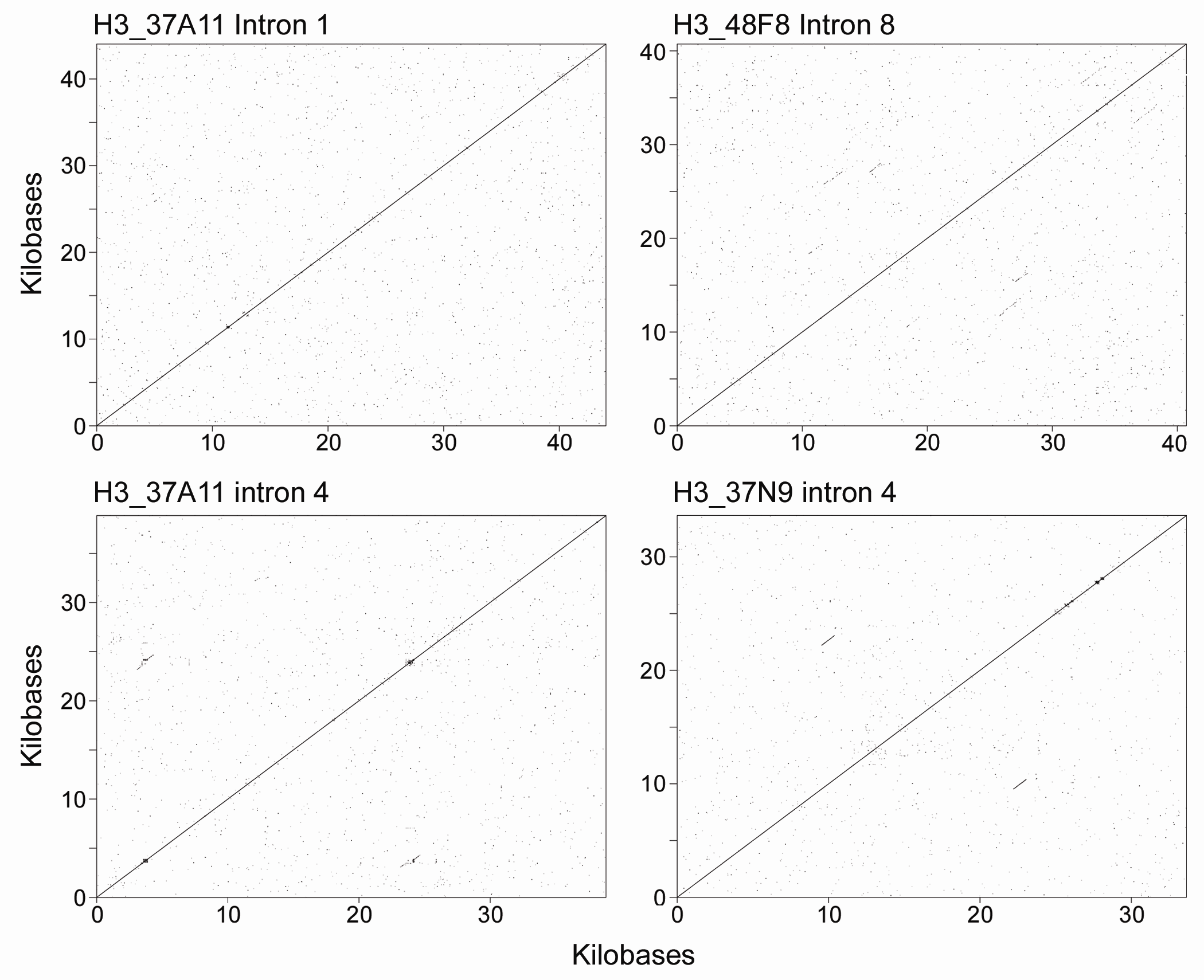

Supplement: Additional file 2 — Self-self sequence alignments. Plots showing representative self-self sequence alignments for the 4 longest introns that were isolated from A. mexicanum. [file 1471-2164-10-19-S2.doc]
