# Supplementary material for: Genic regions of a large salamander genome contain long introns and novel genes
Source: BMC Genomics. 2009 Jan 13;10:19. doi: 10.1186/1471-2164-10-19 (PMC2633012; doi:10.1186/1471-2164-10-19)

**Additional file 3 –** Plot showing the self-alignment of concatenated intronic sequence sampled from *A. mexicanum*. The X and Y axes represent the position along the sequence. The relative location of all alignments >20bp are shown. Repeat sequences should appear as diagonal lines off of the primary diagonal.


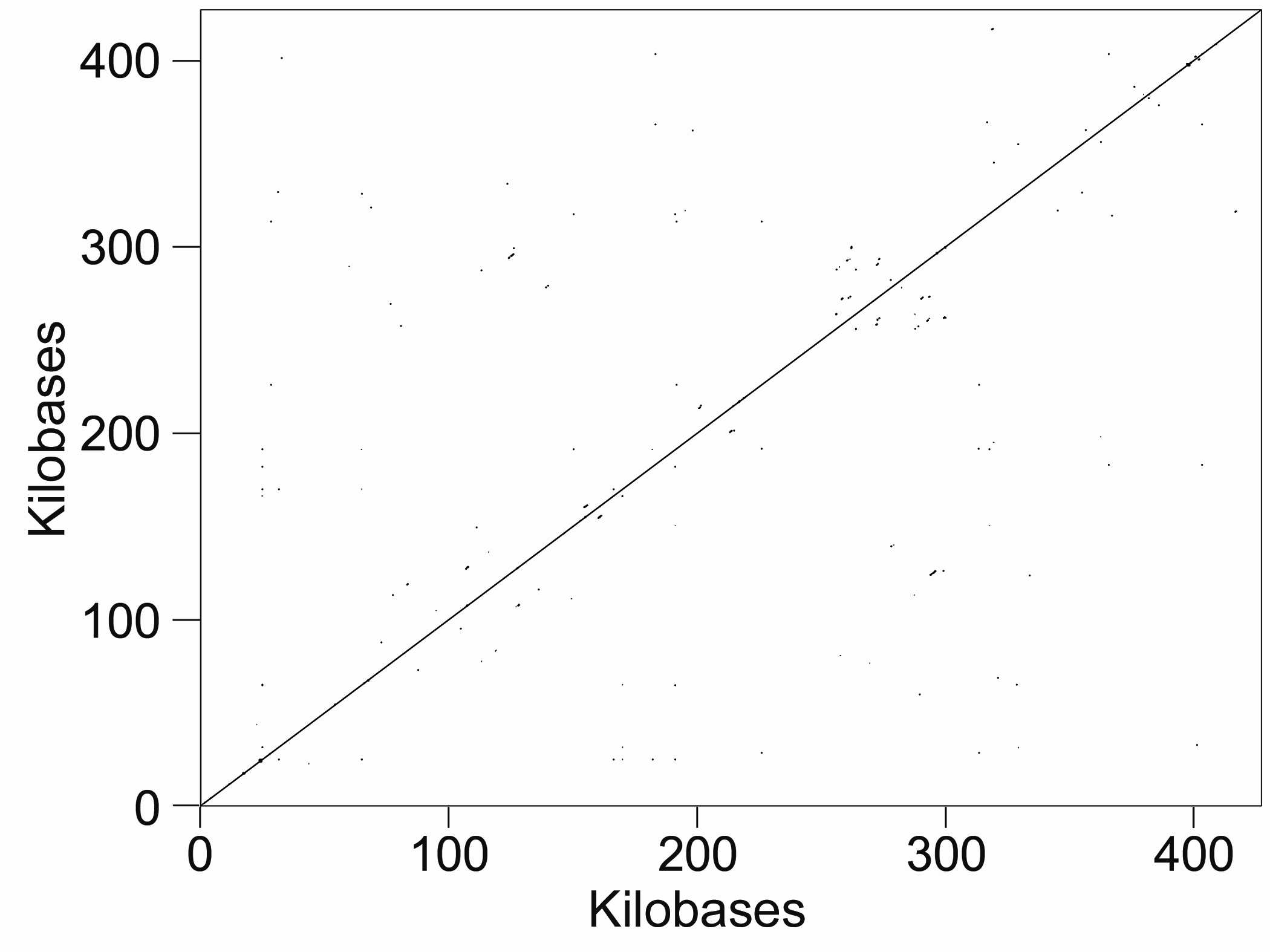

Supplement: Additional file 3 — Alignment of all salamander introns. A plot showing the self-alignment of concatenated intronic sequence sampled from A. mexicanum. [file 1471-2164-10-19-S3.doc]
